# Large scale application of the Finnish diabetes risk score in Latin American and Caribbean populations: a descriptive study

**DOI:** 10.3389/fendo.2023.1188784

**Published:** 2023-06-26

**Authors:** Ramfis Nieto-Martinez, Noël C. Barengo, Manuela Restrepo, Augusto Grinspan, Aria Assefi, Jeffrey I. Mechanick

**Affiliations:** ^1^ Departments of Global Health and Population and Epidemiology, Harvard TH Chan School of Public Health, Boston, MA, United States; ^2^ Precision Care Clinic Corp., Saint Cloud, FL, United States; ^3^ Foundation for Clinic, Public Health, Epidemiology Research of Venezuela (FISPEVEN INC), Caracas, Venezuela; ^4^ Department of Translational Medicine, Herbert Wertheim College of Medicine & Department of Global Health, Robert Stempel College of Public Health and Social Work, Florida International University, Miami, FL, United States; ^5^ Faculty of Medicine, Riga Stradiņš University, Riga, Latvia; ^6^ Medical Affairs Latin America, Merck Kommanditgesellschaft auf Aktien (KGaA), Darmstadt, Germany; ^7^ The Marie-Josée and Henry R. Kravis Center for Cardiovascular Health at Mount Sinai Heart, Division of Endocrinology, Diabetes and Bone Disease, Icahn School of Medicine at Mount Sinai, New York, NY, United States

**Keywords:** glucose metabolism, epidemiology, diabetes screening, dissemination, diabetes risk assessment

## Abstract

**Background:**

The prevalence of type 2 diabetes (T2D) continues to increase in the Americas. Identifying people at risk for T2D is critical to the prevention of T2D complications, especially cardiovascular disease. This study gauges the ability to implement large population-based organized screening campaigns in 19 Latin American and Caribbean countries to detect people at risk for T2D using the Finnish Diabetes Risk Score (FINDRISC).

**Methods:**

This cross-sectional descriptive analysis uses data collected in a sample of men and women 18 years of age or older who completed FINDRISC *via* eHealth during a Guinness World Record attempt campaign between October 25 and November 1, 2021. FINDRISC is a non-invasive screening tool based on age, body mass index, waist circumference, physical activity, daily intake of fruits and vegetables, history of hyperglycemia, history of antihypertensive drug treatment, and family history of T2D, assigning a score ranging from 0 to 26 points. A cut-off point of ≥ 12 points was considered as high risk for T2D.

**Results:**

The final sample size consisted of 29,662 women (63%) and 17,605 men (27%). In total, 35% of subjects were at risk of T2D. The highest frequency rates (FINDRISC ≥ 12) were observed in Chile (39%), Central America (36.4%), and Peru (36.1%). Chile also had the highest proportion of people having a FINDRISC ≥15 points (25%), whereas the lowest was observed in Colombia (11.3%).

**Conclusions:**

FINDRISC can be easily implemented *via* eHealth technology over social networks in Latin American and Caribbean populations to detect people with high risk for T2D. Primary healthcare strategies are needed to perform T2D organized screening to deliver early, accessible, culturally sensitive, and sustainable interventions to prevent sequelae of T2D, and reduce the clinical and economic burden of cardiometabolic-based chronic disease.

## Introduction

The prevalence of type 2 diabetes (T2D) continues to increase in the Americas (1) and worldwide. Recent estimates by the International Diabetes Federation have revealed that 537 million people were living with diabetes in 2021 and this number will most likely increase by 46%, reaching 784 million by 2045 (2). Additionally, all Latin American and Caribbean countries exhibited an increased proportion of all-cause mortality attributable to T2D in the last 30 years (by ~4.7% in men and ~4.8% in women) (3). Arguably, the most troubling aspect of this situation is that many people with T2D are not even aware of their condition; for example, in South and Central America, one out of three patients with diabetes is currently undiagnosed (4). Identification of people with prediabetes or early T2D has been one of the great challenges of modern medicine and reconciling prediabetes as a distinct component of this chronic disease state has been controversial, and at times, even contentious (5) . Though the evidence affirms critical roles of intensive lifestyle change and pharmacotherapy (6–8), large-scale implementation of a formal preventive care approach to mitigating insulin resistance, hyperglycemia, and their respective complications has been elusive.

The dysglycemia-based chronic disease (DBCD) model constitutes a new framework for prevention in the cardiometabolic space. This model comprises 4 stages: stage 1-risk (insulin resistance), stage 2-predisease (prediabetes), stage 3-disease (T2D), and stage 4-complications (vascular disease) (9). The current DBCD model has evolved over the last few years and represents but one of 3 dimensions (i.e., stages, drivers, and social/transcultural determinants), and but one of 5 drivers (the others are abnormal adiposity, hypertension, dyslipidemia, and residual factors such as inflammation) of cardiometabolic-based chronic disease (CMBCD) (10–13). By adopting the DBCD model, a formal culturally adapted, preventive care paradigm can be applied at earlier stages to decrease chronic disease progression and mitigate clinical and economic burdens (9, 14). Pragmatically, insulin resistance and prediabetes are actionable opportunities for early detection to initialize this preventive care process. Fortunately, risk scores have been established as practical and cost-effective tools (15) to identify people at risk for T2D, which could then prompt guideline-directed diagnostic testing, followed by lifestyle change and/or judicious pharmacotherapy/procedures (16, 17).

The Finnish Diabetes Risk Score (FINDRISC) is composed of eight easy-to-collect variables and is the most popular screening tool worldwide (18). The sensitivity and specificity of the FINDRISC to predict 10-year risk of drug-treated T2D are 78-81% and 76-77%, respectively (18). The FINDRISC also identifies patients with abnormal glucose tolerance and occult T2D (19). Of particular importance, the FINDRISC has been applied in several countries and distinct cultures, such as Colombia (20, 21), Venezuela (22), Peru (23), Uruguay (24), Brazil (25), Germany (26), New Zealand (27), U.S (28, 29)., Belgium (30) Spain (31), Greece (32), Jordan (33), Poland (34), Malaysia (35), Turkey (36), Lebanon (25), Norway (37), Sweden (38), Indonesia (39), and aggregated medical practices in Europe (40), leading to the development of population-specific T2D screening. A version of the FINDRISC using specific cutoffs for waist circumference (WC) for the Latino population has also been validated (21, 41) and performs similarly to other FINDRISC versions (23, 42). Most studies describe the accuracy of T2D risk scores for specific populations, but not the implementation logistics in populations at risk (21–23, 29, 41, 43).

Telehealth and social networking have accelerated the implementation of screening tools during the COVID-19 pandemic and can be applied to preventive care plans for chronic metabolic diseases (44). However, relatively few studies have been published on the results of implementing T2D risk scores in large populations (45–49). Even though the FINDRISC has been successfully implemented in several primary healthcare systems (19, 40, 47, 50), this study aims to identify people at high risk of T2D using the FINDRISC through a large population-based telehealth campaign performed in 19 Latin American and Caribbean countries.

## Material and methods

### Study design and population

This cross-sectional descriptive study included a non-probabilistic sample of men and women 18 years of age or older who agreed to complete the FINDRISC on an eHealth platform exclusively available for the period of data collection. Digital surveys were carried out to comply with a Guinness World Record (GWR) attempt campaign entitled “Most digital T2D screening forms collected in 1 week” between October 25 and November 1, 2021 (Brasilia time). To obtain auditable results, the study website including terms, conditions, and privacy policies required management by a third-party data manager. To verify that the methodology was fulfilled (i.e., the surveys corresponded to the FINDRISC questionnaire and users only completed the questionnaire once), two external auditors, one from the medical area and the other from the digital area, were required by the GWR campaign.

The campaign was conducted in 19 countries in North America (Mexico), Central America (Guatemala, El Salvador, Honduras, Nicaragua, Costa Rica, Panama, Dominican Republic, Jamaica, Trinidad and Tobago, Bahamas, Barbados, Aruba, and Curaçao), and South America (Colombia, Chile, Ecuador, Brazil, and Peru). In each country, the FINDRISC was disseminated through press releases and social networks (Instagram® and Facebook®). In some countries, influencers made the link to the website known to their followers. Others interested in participating received the website link to complete the FINDRISC. Once entering the website, participants selected the country of residence, accepted the terms and conditions, and registered their name, last name, and e-mail. User data was protected by confidentiality terms. The data manager performed the database cleaning, eliminating repetitions and inconsistencies. Surveys in which the user did not accept the terms and conditions, did not answer all the questions, or made multiple entries were excluded. The social media channel proviuded constant metrics regarding the usage of the questionnaire through google and meta-analytics. The Guinness records organization demanded an independent platform to manage the metrics for this record attempt. That platform was specifically designed to pull the data from google and meta-analytics, so we did not have any influence in its results during the week the screening campaign was performed. Furthermore, the screening platform was managed by an independent agency.

### Assessing type 2 diabetes risk

The FINRISC is a non-invasive tool that assigns a score from 0 to 26 points to estimate the T2D risk. FINDRISC was translated into Spanish (www.unrecordporlasalud.com), Portuguese (www.umrecordepelasaude.com), and English (www.arecordforheatlh.com). FINDRISC variable definitions and categories are summarized in **Table 1**.

**Table 1:** Categorization and definitions of FINDRISC components.

FINDRISC T2D risk categories^1^	Score	Risk^2^ (%)
Low	0-6	1
Mild	7-11	4
Middle	12-14	17
High	15-20	33
Very high	21-26	50
FINDRISC variables	Variable categories	Score^1^
Age	<45	0
	≥ 45 to < 55	2
	≥ 55 to < 65	3
	≥ 65	4
BMI (kg/m^2^)	Normal (<25)	0
	Overweight (≥ 25 to < 30)	1
	Obesity (≥ 30)	3
WC (cm)	Normal (Men, < 94; women, < 80)	0
	Moderately high WC (Men, ≥ 94 to < 102; women, ≥ 80 to < 88)	3
	Abdominal obesity (Men, ≥ 102, women ≥ 88)	4
≥ 30 min of physical activity/day	No	0
	Yes	2
Daily vegetables/fruits intake	No	0
	Yes	1
Use of blood pressure medication	No	0
	Yes	2
History of high blood glucose	No	0
	Yes	5
Family history of diabetes	No	0
	Yes (second degree relatives^3^)	3
	Yes (first degree relatives^4^)	5
Maximum total score		26

^1^Data from the original FINDRISC study (18). ^2^Risk to develop T2D in the next 10 years. ^3^Second degree relatives include grandparents, aunt, uncle or first cousin. ^4^First degree relatives include parents, brother, sister, or own child.

BMI, Body mass index; FINDRISC, Finnish Diabetes Risk Score; T2D, type 2 diabetes; WC, waist circumference.

### Selection of the FINDRISC cutoffs

The results of studies using, validating, and adapting the FINDRISC in Latin America to identify people with unknown T2D or at risk for T2D (prediabetes: impaired fasting glucose and/or impaired glucose tolerance) is given in **Table 2**. Most studies used the Latin American FINDRISC (LA-FINDRISC), a modified version that applied specific WC cutoffs for the Latino population and compared them with the original FINDRISC (21, 22, 24, 41). The cut-off level used when applying the LA-FINDRISC (21) or a modified FINDRISC (20) to identify people with previously unknown T2D within the clinical setting was 14 points. However, thresholds as low as 10 points were applied in some studies that used the FINDRISC to screen the general population for undetected T2D (21, 22). A cut-off point of ≥ 12 points was considered as being at high risk of T2D and therefore needing diagnostics tests. This threshold was a consensual cutoff recommended in most Latin American countries.

**Table 2:** Validation of FINDRISC in Latin America to identify people with previously unknown prediabetes and type 2 diabetes.

Risk Score	Country / Year	Author / Reference	Population setting	n	Diagnostic test	Aim	Sensitivity/ Specificity (%)	AUC-ROC	Cut-off to detect IGT or uT2D
Original FINDRISC	Finland/2003	Lindstrom and Tuomilehto (7, 18)	General population	4,435	OGTT	Determine if T2D can be prevented by lifestyle interventions in subjects at high risk for the disease	O-FINDRISC Cohort (1987) 78/81 Cohort (1992) 77/76	O-FINDRISC Cohort (1987) 0.85 Cohort (1992) 0.87	≥ 9
LA- FINDRISC	Colombia/ 2012	Aschner et al (41)	General population	421	OGTT	Compare LA- FINDRISC vs O-FINDRISC	LA-FINDRISC Men 74/60 Women 77/67	LA-FINDRISC Men 0.77 Women 0.78	> 12
	Venezuela/ 2012	Aschner et al (41)	Clinical	334	OGTT	Compare LA-FINDRISC vs O-FINDRISC	LA-FINDRISC Men 97/70 Women 91/78	LA-FINDRISC Men 0.91 Women: 0.92	> 14
	Uruguay/ 2015	Vignoli et al (24)	Clinical	109	OGTT	Evaluate LA- FINDRISC performance	LA-FINDRISC 70/66	LA-FINDRISC Overall 0.74	> 14
	Venezuela/ 2015	Nieto-Martínez et al. (22)	General population (National)	3,061	OGTT	Compare LA-FINDRISC vs O-FINDRISC	LA-FINDRISC For uT2D: Men 72/62; Women 71/65 For IGT: Men 65/63 Women 64/62	LA-FINDRISC For uT2D: Men 0.72 Women 0.72 For IGT: Men 0.69 Women 0.67	For uT2D ≥ 9 men ≥ 10 women For IGT ≥ 9 (both sexes)
ColDRISC*	Colombia/ 2015	Barengo et al. (51)	Captive population (insurance company)	2,060	OGTT	Develop and compare ColDRISC vs LA-FINDRISC	ColDRISC 73/67 LA-FINDRISC 72/60	ColDRISC 0.74 LA-FINDRISC 0.73	ColDRISC > 4
Modified FINDRISC	Colombia/2015	Gomez-Arbelaez et al. (20)	Clinical	772	A1C	To evaluate the performance of FINDRISC detecting and predicting T2D	Modified-FINDRISC Men 66/75 Women 71/62	Modified-FINDRISC Men 0.74 Women 0.71	> 14
Peruvian “simplified” Risk Score*	Peru/2018	Bernabe-Ortiz et al (23)	General population	1,609	OGTT	Compare O-FINDRISC, LA-FINDRISC, and Peruvian Risk Score, and derived Simplified FINDRISC version		O-FINDRISC 0.69, LA-FINDRISC 0.68 Peruvian Risk Score 0.64 Simplified FINDRISC 0.71	

A1C, Glycated hemoglobin A1C; AUC, area under the curve; IFG, impaired fasting blood glucose; IGR, Impaired glucose regulation;, IGT, impaired glucose tolerance; O-FINDRISC, Original Finnish Diabetes Risk Score; OGTT, Oral glucose tolerance test; ROC, receiver-operating characteristic; T2D, type 2 diabetes; uT2D, unknown type 2 diabetes. FINDRISC versions: (1) ColDRISC: Colombian Diabetes Risk Score, (2) LA-FINDRISC: Latin America FINDRISC, (3) Modified FINDRISC: include a modification in the WC cut-off values: Men: < 90 cm (0 risk points); 90-98 cm (3 risk points); > 98 cm (4 risk points). Women: < 80 cm, (0 risk points); 80-88 cm (3 risk points); > 88 cm (4 risk points). *Score is not comparable with the O-FINDRISC version.

### Statistical analysis

Data were analyzed using SPSS 20 software (IBM corp. Released 2011; Armonk, NY, USA). Frequencies were presented as percentages and 95% confidence intervals (CI), and differences between groups were considered when no 95% CI overlap was detected. A p-value of <0.05 was considered statistically significant. All procedures were performed in accordance with the Helsinki Declaration. This study was considered as Non-Human Subject Research and therefore not requiring Institutional Review Board approval.

## Results

### Subjects’ characteristics

The final sample size comprised 47,267 subjects from 19 countries, comprising 13 Central American countries (merged for the analysis and reported as the Central America region), Mexico, Colombia, Chile, Ecuador, Brazil, and Peru. Of the total sample, 62.8% were women with a mean age of 48 ± 0.02 (mean ± SE), 86.8% were < 55 years of age, and 89.4% were from Brazil, Mexico, or Peru. Compared with the women, the men were older (+ 9% of subjects ≥ 45 years), with a higher proportion of overweight (+ 11.9%), less daily intake of fruits and vegetables (-1.8%), and greater use of blood pressure medications (+7.1%). Compared with the men, the women had a higher proportion of abdominal obesity (+ 9.5%), physical inactivity (+ 12.3%), personal history of high blood glucose (+ 1.1%), and second-degree relatives with a history of T2D (+ 4.6%) (**Table 3**).

**Table 3:** Characteristics of study subjects by sex.

	Total	Men	Women
	n (%, 95%CI)	n (%, 95%CI)	n (%, 95%CI)
Total	47267	17605 (37.2, 36.8 - 37.7)	29662 (62.8, 62.3 - 63.2)
Countries			
Brazil	21925 (46.4, 45.9-46.8)	8899 (50.5, 49.8 - 51.3)	13026 (43.9, 43.4 - 44.5)
Mexico	15264 (32.3, 31.9-32.7)	5823 (33.1, 32.4 - 33.8)	9441 (31.8, 31.3 - 32.4)
Peru	5059 (10.7, 10.4-11.0)	1049 (6.0, 5.6 - 6.3)	4010 (13.5, 13.1 - 13.9)
Colombia	2331 (4.9, 4.7-5.1)	912 (5.2, 4.9 - 5.5)	1419 (4.8, 4.5 - 5.0)
Central America	1117 (2.4, 2.2-2.5)	434 (2.5, 2.2 - 2.7)	683 (2.3, 2.1 - 2.5)
Ecuador	663 (1.4, 1.3-1.5)	234 (1.3, 1.2 - 1.5)	429 (1.4, 1.3 - 1.6)
Chile	520 (1.1, 1.0-1.2)	171 (1.0, 0.8 - 1.1)	349 (1.2, 1.1 - 1.3)
	% (95% CI)	% (95% CI)	% (95% CI)
Age (years)			
<45	66.7 (66.3-67.1)	61.1 (60.4-61.8)	70.0 (69.5-70.5)
≥ 45 to < 55	20.1 (19.7-20.4)	22.7 (22.1-23.3)	18.5 (18.0-18.9)
≥ 55 to < 65	9.9 (9.7-10.2)	12.1 (11.6-12.6)	8.6 (8.3-8.9)
≥ 65	3.3 (3.2-3.5)	4.1 (3.8-4.4)	2.9 (2.7-3.1)
BMI (kg/m^2^)			
Normal weight (<25)	36.4 (35.9-36.8)	28.5 (27.9-29.2)	41.0 (40.5-41.6)
Overweight (≥ 25 to < 30)	37.2 (36.7-37.6)	44.6 (43.9-45.4)	32.7 (32.2-33.3)
Obesity (≥ 30)	26.4 (26.1-26.9)	26.9 (26.2-27.5)	26.3 (25.8-26.8)
WC (cm)			
Normal (Men, < 94; women, < 80)	48.8 (48.4-49.3)	52.2 (51.5-53.0)	46.8 (46.2-47.3)
Moderately high WC (Men, ≥ 94 to < 102; women, ≥ 80 to < 88)	32.4 (32.0-32.8)	35.0 (34.3-35.7)	30.9 (30.4-31.4)
Abdominal obesity (Men, ≥ 102, women ≥ 88)	18.8 (18.4-19.1)	12.8 (12.3-13.3)	22.3 (21.9-22.8)
≥ 30 min of physical activity/day (no)	57.3 (56.9-57.8)	49.6 (48.9-50.4)	61.9 (61.3-62.4)
Daily vegetables/fruits intake (no)	40.8 (40.3-41.2)	41.9 (41.2-42.7)	40.1 (39.5-40.7)
Use of blood pressure medication (yes)	18.9 (18.5-19.2)	23.3 (22.7-23.9)	16.2 (15.8-16.7)
History of high blood glucose (yes)	22.5 (22.1-22.9)	21.8 (21.2-22.4)	22.9 (22.4-23.4)
Family history of diabetes			
No	30.0 (29.6-30.4)	33.3 (32.7-34.0)	28.0 (27.5-28.5)
Second degree relatives^1^ (yes)	39.0 (38.6-39.5)	36.1 (35.4-36.8)	40.7 (40.2-41.3)
First degree relatives^2^ (yes)	31.0 (30.6-31.4)	30.6 (29.8-31.2)	31.3 (30.7-31.8)
FINDRISC score ≥ 12	34.7 (34.3-35.2)	33.7 (33.0-34.4)	35.4 (34.8-35.9)
FINDRISC score ≥ 15	18.5 (18.2-18.9)	18.3 (17.8-18.9)	18.7 (18.2-19.1)

Frequencies are expressed as percentages and 95% CI and differences were considered when no 95% CI overlap was detected. ^1^Second degree relatives include grandparents, aunt, uncle or first cousin. ^2^First degree relatives include parents, brother, sister, or own child.

BMI, Body mass index; CI, Confidence Interval; FINDRISC, Finnish Diabetes Risk Score; WC, Waist circumference.

### FINDRISC components by T2D risk categories

Overall, 33% the subjects were at low risk to develop T2D (FINDRISC < 7), 32.3% at slightly elevated risk (FINDRISC 7-11), 16.2% at moderate risk (FINDRISC 12-14), 15.5% at high risk (FINDRISC 15-20), and 3.0% at very high risk (FINDRISC > 20) (**Table 4**). The risk of T2D increased with age, adiposity, physical inactivity, low intake of fruits and vegetables, use of blood pressure medications, history of hyperglycemia, and family history of T2D. Although 42.9% of the youngest population (< 45 years of age) had a low risk (FINDRISC < 7), 9.9% of them had a high risk of T2D (FINDRISC 15-20). The proportion of subjects at high risk of T2D increased in each decade of age reaching 34.0% in those ≥ 65 years old.

**Table 4:** Distribution of FINDRISC components by T2D risk categories.

FINDRISC categories Risk to develop T2D in the next 10 years	< 7 Low (1%)	7-11 Mild (4%)	12-14 Middle (17%)	15-20 High (33%)	>20 Very high (50%)
Total (%, 95%CI)	33.0 (32.6 - 33.4)	32.3 (31.9 - 32.7)	16.2 (15.8 - 16.5)	15.5 (15.2 - 15.9)	3.0 (2.8 - 3.2)
Age (years)					
<45	42.9 (42.4 - 43.5)	32.7 (32.2 - 33.2)	13.8 (13.4 - 14.1)	9.9 (9.5 - 10.2)	0.7 (0.6 - 0.8)
≥ 45 to < 55	15.3 (14.6 - 16.1)	34.3 (33.4 - 35.3)	21.2 (20.4 - 22.0)	23.6 (22.8 - 24.5)	5.6 (5.1 - 6.0)
≥ 55 to < 65	10.3 (9.5 - 11.2)	27.9 (26.7 - 29.2)	20.8 (19.7 - 22.0)	31.3 (29.9 - 32.5)	9.7 (8.9 - 10.6)
≥ 65	6.9 (5.7 - 8.2)	24.3 (22.3 - 26.5)	20.8 (18.8 - 22.8)	34.0 (31.8 - 36.5)	14.0 (12.3 - 15.8)
BMI (kg/m^2^)					
Normal (<25)	60.2 (59.4 - 60.9)	28.9 (28.2 - 29.6)	7.0 (6.7 - 7.4)	3.6 (3.4 - 3.9)	0.3 (0.2 - 0.4)
Overweight (≥ 25 to < 30)	26.6 (25.9 - 27.2)	41.0 (40.2 - 41.7)	17.0 (16.5 - 17.6)	13.9 (13.4 - 14.5)	1.5 (1.3 - 1.7)
Obese (≥ 30)	4.7 (4.3 - 5.1)	24.8 (24.0 - 25.5)	27.5 (26.8 - 28.3)	34.2 (33.4 - 35.0)	8.8 (8.3 - 9.3)
WC (cm)					
Normal (Men, < 94; women, < 80)	60.3 (59.7 - 60.9)	29.7 (29.1 - 30.3)	6.8 (6.4 - 7.1)	3.0 (2.8 - 3.3)	0.2 (0.2 - 0.3)
Moderately high WC (Men, ≥ 94 to < 102; women, ≥ 80 to < 88)	10.2 (9.8 - 10.7)	41.8 (41.0 - 42.6)	24.0 (23.3 - 24.7)	20.8 (20.1 - 21.4)	3.2 (3.0 - 3.5)
Abdominal obesity (Men, ≥ 102, women ≥ 88)	1.3 (1.1 - 1.5)	22.7 (21.8 - 23.5)	27.2 (26.3 - 28.2)	39.0 (38.0 - 40.1)	9.8 (9.2 - 10.4)
≥ 30 min of physical activity/day					
No	21.8 (21.3 - 22.3)	33.7 (33.2 - 34.3)	19.5 (19.0 - 20.0)	20.4 (19.9 - 20.9)	4.6 (4.4 - 4.9)
Yes	48.1 (47.3 - 48.7)	30.4 (29.7 - 31.0)	11.7 (11.3 - 12.2)	9.0 (8.7 - 9.4)	0.8 (0.7 - 1.0)
Daily vegetables/fruits intake					
No	24.7 (24.1 - 25.3)	33.7 (33.0 - 34.3)	18.8 (18.3 - 19.4)	19.2 (18.6 - 19.7)	3.6 (3.3 - 3.9)
Yes	38.7 (38.1 - 39.3)	31.3 (30.8 - 31.9)	14.3 (13.9 - 14.7)	13.1 (12.7 - 13.5)	2.6 (2.4 - 2.8)
Use of blood pressure medication					
No	39.0 (38.5 - 39.5)	34.0 (33.6 - 34.5)	15.1 (14.7 - 15.4)	11.0 (10.7 - 11.3)	0.9 (0.8 - 1.0)
Yes	7.0 (6.5 - 7.6)	24.8 (23.9 - 25.7)	21.0 (20.2 - 21.9)	35.2 (34.2 - 36.1)	12.0 (11.3 - 12.7)
History of high blood glucose					
No	42.0 (41.5 - 42.6)	36.3 (35.8 - 36.8)	14.7 (14.3 - 15.0)	6.8 (6.5 - 7.0)	0.2 (0.2 - 0.3)
Yes	1.7 (1.5 - 2.0)	18.6 (17.9 - 19.4)	21.3 (20.6 - 22.1)	45.8 (44.9 - 46.8)	12.6 (11.9 - 13.2)
Family history of diabetes					
No	57.5 (56.7 - 58.3)	29.8 (29.0 - 30.5)	7.7 (7.3 - 8.1)	4.8 (4.5 - 5.2)	0.2 (0.1 - 0.3)
Second degree relatives^1^ (yes)	33.9 (33.3 - 34.6)	34.5 (33.8 - 35.2)	17.1 (16.6 - 17.6)	13.0 (12.6 - 13.5)	1.5 (1.3 - 1.6)
First degree relatives^2^ (yes)	8.1 (7.6 - 8.5)	32.0 (31.2 - 32.7)	23.2 (22.6 - 23.9)	29.1 (28.4 - 29.8)	7.6 (7.2 - 8.1)

Frequencies are expressed as percentages and 95% CI and differences were considered when no 95% CI overlap was detected. ^1^Second degree relatives include grandparents, aunt, uncle or first cousin. ^2^First degree relatives include parents, brother, sister, or own child.

BMI, Body mass index; CI, Confidence Interval; FINDRISC, Finnish Diabetes Risk Score; T2D, type 2 diabetes; WC, waist circumference.

The risk of T2D was low or slightly elevated (FINDRISC < 12) in 89.1% of subjects with normal weight and 90% of subjects without abdominal obesity. Excess total (by BMI) and central (by WC) adiposity increased the risk of T2D. Compared with subjects with normal weight (3.6%), the high risk of T2D (FINDRISC 15-20) increased to 13.9% in subjects with overweight and 34.2% in those with obesity, and the proportion of subjects with very high risk (FINDRISC > 20) increased 5-fold with overweight and almost 30-fold with obesity. Compared with normal WC (3.0%), a high risk of T2D (FINDRISC 15-20) increased to 20.8% in subjects with moderately-high WC and to 39.0% in those with abdominal obesity, and the proportion of subjects with very high risk (FINDRISC > 20) increased 16 times with a moderate increase in WC and almost 50 times with the presence of abdominal obesity (**Table 4**).

Almost 80% of subjects with a FINDRISC < 12 reported participating in ≥ 30 min of physical activity/day compared to less than 1% in the very high-risk group (score > 20). Likewise, 70% of those with low-mild risk of T2D reported that they consumed fruits and vegetables daily compared to only 2.6% in the very high-risk group. Sixty-eight percent of subjects with FINDRISC ≥ 12 reported using blood pressure medication. In the T2D high risk and very-high risk groups, the use of BP medication was 3 and 13 times higher, respectively. A personal history of hyperglycemia was reported by 80% of subjects with a FINDRISC ≥ 12. In the groups with high and very-high risk for T2D, a history of hyperglycemia was 7 and 63 times higher, respectively. Sixty percent and 31.6% of subjects with a FINDRISC ≥ 12 reported first and second-degree relatives with T2D, respectively, whereas only 12.7% did not (**Table 4**).

### T2D risk in different countries/regions

**Figure 1:**
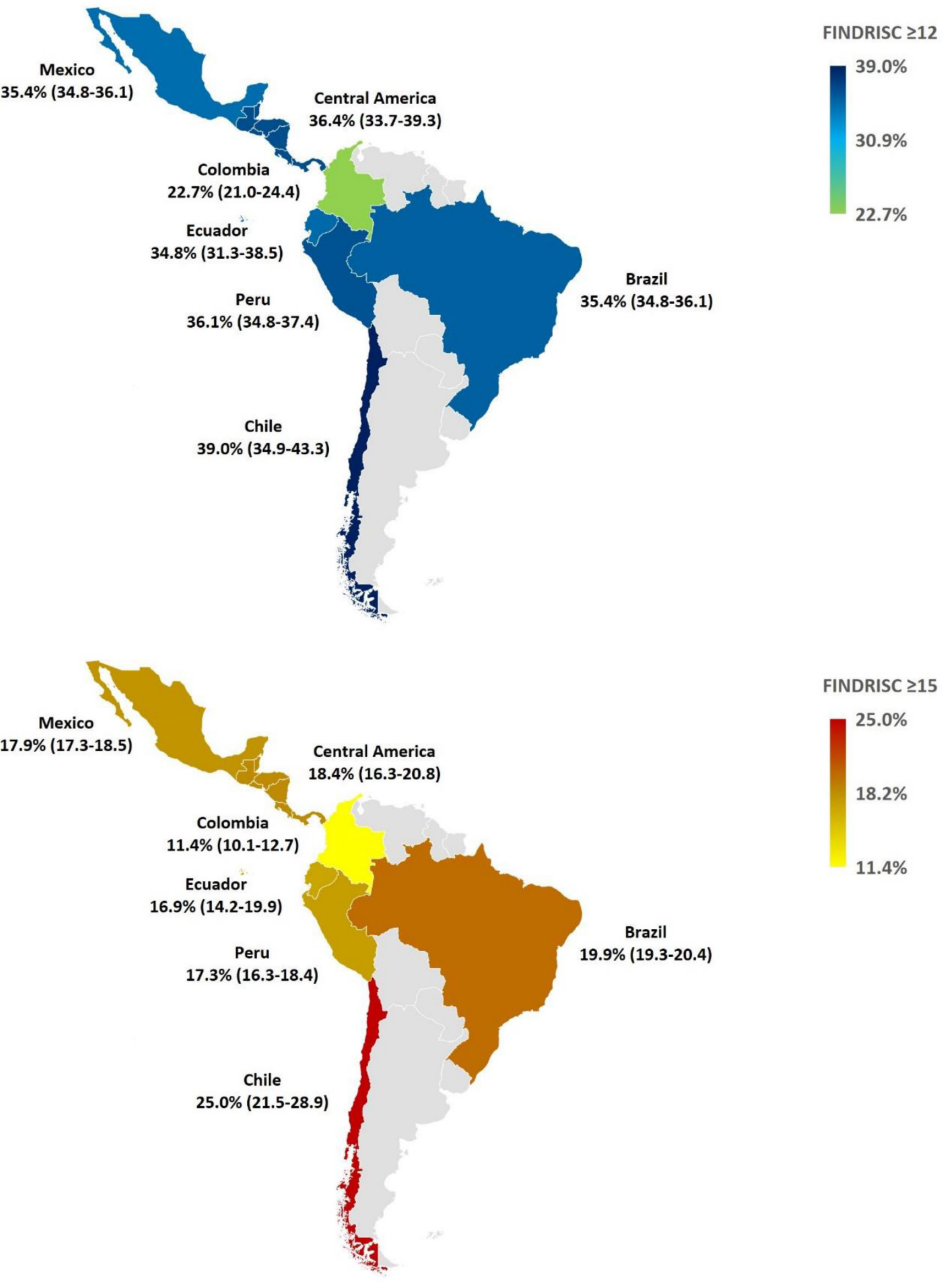
Geographical map of FINDRISC scores ≥12 points (upper) and ≥15 points (bottom) by country/region. Frequencies are expressed as percentages and 95% CI ≥12 points (upper) and ≥15 points (bottom) by country. Differences were considered when no 95% CI overlap was detected. Data from Guatemala, El Salvador, Honduras, Nicaragua, Costa Rica, Panama, Dominican Republic, Jamaica, Trinidad and Tobago, Bahamas, Barbados, Aruba, and Curaçao were aggregated as Central America region. CI, Confidence Interval; FINDRISC, Finnish Diabetes Risk Score.

In total, 34.5% and 18.5% of all study subjects reported a FINDRISC of at least 12 points and 15 points respectively (**Table 3**; **Figure 1**), which provides an approximate number of people at risk of T2D in the countries surveyed. No differences by sex were found (**Table 3**). Using a cutoff of ≥ 12 points, the risk of T2D was similar in all studied countries varying from 34.4% in Ecuador to 39% in Chile, but lowest in Colombia (22.7%) than the rest of the countries. Using a cutoff of ≥ 15 points, the risk of T2D was similar in all countries ranging from 16.9% in Ecuador to 19.9% in Brazil, but lowest in Colombia (11.4%) and highest in Chile (25%), compared with the rest of the countries (**Figure 1**).

More than 80% of subjects were under 55 years of age. The youngest population was in Peru (96% < 55 y), whereas the oldest were in Brazil (18.0% ≥ 55 y). The prevalence of obesity was highest in Central America (37.7%), and lowest in Colombia (15.1%). Abdominal obesity was most prevalent in Peru (30.6%) and least prevalent in Colombia (10.2%). Reporting at least 30 minutes of physical activity was highest in Colombia (51.8%) and lowest in Peru (30.7%); whereas daily intake of fruits and vegetables was most prevalent in Brazil (66.1%) and least prevalent in Peru (37.7 %) The use of blood pressure medications was similar in all countries (ranging from 17.9% in Mexico to 21.4% in Brazil), except in Peru where it was lowest (9.9%). A personal history of hyperglycemia ranged from 20.1% in Mexico to 33.1% in Chile. A family history of T2D in first degree relatives was highest in Mexico (36.7%) and lowest in Colombia (18.7%) (**Table 5**).

**Table 5:** Distribution of T2D risk and FINDRISC components by studied countries/region.^1^.

	Chile	Brazil	Central America *	Mexico	Peru	Ecuador	Colombia
	% (95% CI)	% (95% CI)	% (95% CI)	% (95% CI)	% (95% CI)	% (95% CI)	% (95% CI)
FINDRISC categories							
< 7 (Low risk)	30.2 (26.4 - 34.3)	33.3 (32.7 - 33.9)	30.7 (28.1 - 33.5)	32.5 (31.7 - 33.2)	28.7 (27.5 - 30.0)	32.4 (29.0 - 36.1)	45.2 (43.2 - 47.2)
7-11 (Mild risk)	30.8 (27.0 - 34.9)	31.3 (30.7 - 31.9)	32.9 (30.2 - 35.7)	32.9 (32.2 - 33.6)	35.2 (33.9 - 36.5)	32.7 (29.3 - 36.4)	32.2 (30.3 - 34.1)
12-14 (Middle risk)	14.0 (11.3 - 17.3)	15.6 (15.1 - 16.1)	18.0 (15.9 - 20.4)	16.7 (16.1 - 17.3)	18.8 (17.7 - 19.9)	17.9 (15.2 - 21.1)	11.3 (10.1 - 12.6)
15-20 (High risk)	21.0 (17.7 - 24.7)	16.0 (15.5 - 16.5)	15.4 (13.4 - 17.6)	15.2 (14.7 - 15.8)	16.2 (15.3 - 17.3)	15.8 (13.1 - 18.7)	10.1 (9.0 - 11.5)
> 20 (Very high risk)	4.0 (2.7 - 6.1)	3.8 (3.6 - 4.1)	3.0 (2.2 - 4.2)	2.7 (2.4 - 2.9)	1.1 (0.8 - 1.4)	1.2 (0.6 - 2.4)	1.2 (0.8 - 1.7)
Age (years)							
<45	63.5 (59.2 - 67.5)	59.8 (59.1 - 60.4)	72.2 (69.5 - 74.7)	67.2 (66.5 - 68.0)	86.7 (85.7 - 87.5)	76.6 (73.3 - 79.7)	78.4 (76.7 - 80.0)
≥ 45 to < 55	25.0 (21.5 - 28.9)	22.2 (21.7 - 22.8)	19.4 (17.2 - 21.9)	21.4 (20.7 - 22.0)	9.3 (8.6 - 10.2)	15.4 (12.8 - 18.3)	15.9 (14.5 - 17.5)
≥ 55 to < 65	9.6 (7.4 - 12.5)	13.2 (12.7 - 13.6)	6.3 (5.0 - 7.8)	8.8 (8.3 - 9.2)	3.2 (2.7 - 3.7)	6.5 (4.9 - 8.6)	4.8 (4.0 - 5.7)
≥ 65	1.9 (1.0 - 3.5)	4.8 (4.6 - 5.1)	2.1 (1.4 - 3.2)	2.6 (2.3 - 2.8)	0.8 (0.6 - 1.1)	1.5 (0.8 - 2.8)	0.9 (0.6 - 1.4)
BMI (kg/m^2^)							
Normal (<25)	36.2 (32.1 - 40.4)	35.0 (34.3 - 35.6)	32.3 (29.6 - 35.1)	37.5 (36.7 - 38.3)	32.1 (30.8 - 33.4)	38.6 (35.0 - 42.4)	53.8 (51.8 - 55.8)
Overweight (≥ 25 to < 30)	34.8 (30.8 - 39.0)	39.4 (38.8 - 40.1)	30.0 (27.4 - 32.7)	37.1 (36.3 - 37.9)	33.3 (32.0 - 34.6)	32.1 (28.7 - 35.8)	31.1 (29.3 - 33.0)
Obesity (≥ 30)	29.0 (25.3 - 33.1)	25.6 (25.0 - 26.2)	37.7 (34.9 - 40.6)	25.4 (24.7 - 26.1)	34.6 (33.3 - 36.0)	29.3 (25.9 - 32.8)	15.1 (13.7 - 16.6)
Waist circumference (cm)							
Normal (Men, < 94; women, < 80)	45.0 (40.8 - 49.3)	49.5 (48.8 - 50.1)	50.3 (47.3 - 53.2)	49.9 (49.1 - 50.7)	38.0 (36.7 - 39.4)	47.1 (43.3 - 50.9)	59.6 (57.6 - 61.6)
Moderately high WC (Men, ≥ 94 to < 102; women, ≥ 80 to < 88)	34.4 (30.5 - 38.6)	32.5 (31.9 - 33.1)	33.0 (30.3 - 35.8)	33.0 (32.2 - 33.7)	31.4 (30.1 - 32.7)	35.3 (31.8 - 39.0)	30.2 (28.4 - 32.1)
Abdominal obesity (Men, ≥ 102, women ≥ 88)	20.6 (17.3 - 24.3)	18.0 (17.5 - 18.5)	16.7 (14.7 - 19.0)	17.1 (16.5 - 17.7)	30.6 (29.3 - 31.9)	17.6 (14.9 - 20.7)	10.2 (9.0 - 11.5)
≥ 30 min of physical activity/day							
No	64.4 (60.2 - 68.4)	58.8 (58.2 - 59.5)	52.2 (49.3 - 55.1)	52.4 (51.6 - 53.2)	69.3 (68.1 - 70.6)	57.2 (53.4 - 60.9)	48.2 (46.2 - 50.2)
Yes	35.6 (31.6 - 39.8)	41.2 (40.5 - 41.8)	47.8 (44.9 - 50.7)	47.6 (46.8 - 48.4)	30.7 (29.4 - 31.9)	42.8 (39.1 - 46.6)	51.8 (49.8 - 53.8)
Daily vegetables/fruits intake							
No	39.4 (35.3 - 43.7)	33.9 (33.3 - 34.5)	49.6 (46.7 - 52.5)	42.1 (41.3 - 42.9)	62.3 (60.9 - 63.6)	49.9 (46.1 - 53.7)	42.1 (40.1 - 44.1)
Yes	60.6 (56.3 - 64.7)	66.1 (65.5 - 66.7)	50.4 (47.5 - 53.3)	57.9 (57.1 - 58.7)	37.7 (36.4 - 39.1)	50.1 (46.3 - 53.9)	57.9 (55.9 - 59.9)
Use of blood pressure medication							
No	80.8 (77.2 - 83.9)	78.6 (78.1 - 79.2)	78.5 (76.0 - 80.8)	82.1 (81.4 - 82.7)	90.1 (89.2 - 90.9)	80.2 (77.0 - 83.1)	80.4 (78.7 - 82.0)
Yes	19.2 (16.1 - 22.8)	21.4 (20.8 - 21.9)	21.5 (19.2 - 24.0)	17.9 (17.3 - 18.6)	9.9 (9.1 - 10.8)	19.8 (16.9 - 23.0)	19.6 (18.0 - 21.3)
History of high blood glucose							
No	66.9 (62.8 - 70.8)	76.5 (75.9 - 77.1)	76.5 (73.9 - 78.8)	79.6 (79.0 - 80.2)	78.3 (77.2 - 79.4)	73.9 (70.4 - 77.1)	75.5 (73.7 - 77.2)
Yes	33.1 (29.2 - 37.2)	23.5 (22.9 - 24.1)	23.5 (21.2 - 26.1)	20.4 (19.8 - 21.0)	21.7 (20.6 - 22.8)	26.1 (22.9 - 29.6)	24.5 (22.8 - 26.3)
Family history of diabetes							
No	35.2 (31.2 - 39.4)	33.0 (32.3 - 33.6)	26.2 (23.7 - 28.9)	22.8 (22.2 - 23.5)	35.0 (33.7 - 36.3)	31.4 (28.0 - 35.0)	38.4 (36.4 - 40.3)
Second degree relatives^2^ (yes)	36.0 (32.0 - 40.2)	37.3 (36.7 - 38.0)	40.7 (37.8 - 43.6)	40.5 (39.7 - 41.2)	39.9 (38.6 - 41.3)	43.9 (40.2 - 47.7)	42.9 (40.9 - 44.9)
First degree relatives^3^ (yes)	28.8 (25.1 - 32.9)	29.7 (29.1 - 30.3)	33.1 (30.4 - 35.9)	36.7 (35.9 - 37.5)	25.1 (23.9 - 26.3)	24.7 (21.6 - 28.2)	18.7 (17.2 - 20.4)

Frequencies are expressed as percentages and 95% CI and differences were considered when no 95% CI overlap was detected. ^1^Data from Guatemala, El Salvador, Honduras, Nicaragua, Costa Rica, Panama, Dominican Republic, Jamaica, Trinidad and Tobago, Bahamas, Barbados, Aruba, and Curaçao were aggregated as Central America region. ^2^Second degree relatives include grandparents, aunt, uncle or first cousin. ^3^First degree relatives include parents, brother, sister, or own child.

BMI, Body mass index; CI, Confidence Interval; FINDRISC, Finnish Diabetes Risk Score; WC, waist circumference.

## Discussion

Large-scale application of the FINDRISC eHealth version as part of an organized screening program to assess risk for T2D was feasible in Latin American and Caribbean populations representing 19 countries. The 47,267 subjects evaluated in 1 week set a Guinness World Record. This study revealed that 35% of the population studied was at risk of T2D, with 1,418 (3%) having a 50% risk for developing T2D in the next 10 years. In this very high-risk group, the risk increased with low fruit and vegetable intake by 1.4 times, low physical activity by 6 times, use of blood pressure medications by 13 times, age > 65 years by 20 times, obesity by 29 times, family history of T2D by 38 times, abdominal obesity by 49 times, and history of hyperglycemia by 63 times.

Applications of FINDRISC scoring in Latin America incorporate specific cut-offs to detect prediabetes, occult T2D, or known T2D (20–24, 41, 42), but few have been leveraged to proactively detect subjects at risk of T2D with the intent of initializing a formal preventive care plan. Diagnostic and prognostic models for T2D among randomly selected adults in Latin America are also scarce (43). Community pharmacy-based opportunistic screening programs are one such example of successful implementation of FINDRISC scoring. In one campaign spanning 854 pharmacies from Spain and Italy, FINDRISCs were collected in 7,234 subjects (52). Of them, 65.5% (vs 65.3% in this study) were at low/slightly elevated risk to develop T2D (FINDRISC < 12), 19.3% (vs 16.2% in this study) were at moderate risk (FINDRISC 12-14), 13.9% (vs 15.5% in this study) were at high risk (FINDRISC 15-20), and 1.4% (vs 3.0% in this study) were at very high risk (FINDRISC > 20). Subjects showing a higher risk of T2D (FINDRISC ≥ 15) in Spain (16.7%) and Italy (14.7%) were lower than Chile (25%), Brazil (19.9%), Central America (18.4%), Mexico (17.9%), Peru (17.3%), and Ecuador (16.9%) in this study, but higher than Colombia (11.4%) (52). A similar campaign performed in 345 municipalities in Brazil involving 977 pharmacists and testing 17,580 subjects between 20 and 79 years found that 22.7% had a high/very high risk of T2D (FINDRISC ≥ 15) (53). This finding is higher than that in the present study in Brazil (19.9%), consistent with a higher risk profile among pharmacy customers compared with eHealth subjects. This is affirmed by an Italian study, in which one-year follow-up after FINDRISC screening of 5,977 community pharmacy customers found that compared with the total sample, those with a FINDRISC ≥ 12 (53% of the total sample) had more fasting blood glucose (FBG; 53.5 vs. 47.8%) and A1C (17.6 vs 12.1%) measurements, as well as evaluations by diabetologists (6.7% vs 5.2%) (54).

Large-scale organized or opportunistic screening to detect patients at risk for T2D should be followed by aggressive case finding, diagnostic testing, lifestyle interventions, and if indicated, pharmaceutical treatment. Using FINDRISC for opportunistic initial screening in 1,377 subjects in Italy followed by FBG measurement in those with a FINDRISC ≥ 9 and then OGTT in those with FBG 100-125 mg/dl, identified 57% with IGT and 83% of cases of T2D (47). Data from 3,866 NHANES subjects showed that the combination of FINDRISC and A1C, compared to FINDRISC alone, improved the sensitivity for detecting T2D from 79.1% to 84.2%, while maintaining similar specificity (48.6% vs 48.3%) (28). In Argentina, combining both organized and opportunistic recruitment, 3,759 individuals completed the FINDRISC, with 43% scoring ≥ 13 points (cutoff selected by expert opinion). This high-risk group then underwent OGTT, detecting 47% with prediabetes (49). A pooled sensitivity and specificity analysis of T2D diagnosis showed that using an A1C-based definition alone will not identify a substantial proportion of previously undiagnosed people who would be considered as having T2D using a glucose-based test; 47.2% less vs FBG, 62.8% less vs OGTT, and 69.6% less vs FBG or OGTT (55). Although the use of A1C for everyone in the T2D care process and creation of infrastructure with this aim has been recommended in various Latin American countries (56), not all laboratories where A1C is measured are properly certified and OGTT could be more accessible and affordable than A1C.

This study elucidates the asymmetric distribution of T2D risk factors among Latin American and Caribbean countries, which has direct impact on public health initiatives such as organized screening, diagnostic testing, and preventive care plans. Except for older subjects in Brazil, younger ones in Peru, and those with a family history of T2D in Mexico, non-modifiable risk factors (i.e., age and family history of T2D) were similar among the countries studied. This indicates that a large part of T2D risk derives from modifiable factors (e.g., adiposity, dysglycemia, hypertension, and eating patterns) that are potentially mitigated by healthy lifestyle change. Notwithstanding a high intake of fruits and vegetables, Chile was the country with the highest risk of T2D (25%) in this study probably related to the high prevalence of abdominal obesity. It should be noted that in Peru, despite having a relatively high proportion of young subjects, had the highest frequency of abdominal obesity, sedentary lifestyle, no daily intake of fruits and vegetables, and the second highest frequency of obesity. The highest proportion of obesity was found in Central America (37.7%) where the proportion of high T2D risk was 18.4%. In contrast, Colombia was the country with the lowest T2D risk (11.4%) and commensurately lowest obesity, abdominal obesity, and sedentary lifestyle prevalence rates. A systematic review that included five population-based studies in three LA countries (Mexico, Brazil, and Peru) found that the most common predictors of T2D were age, WC, and family history of diabetes (43). Using the β-coefficients of the original FINDRISC model, it is estimated that 54% of the FINDRISC score is attributed to modifiable risk factors (18), and of these, almost 80% is related to increased adiposity amount. The implication here is that prevention imperatives to reduce T2D risk should prioritize weight reduction tactics. In 1079 subjects receiving lifestyle intervention and followed for a mean of 3.2 years in the Diabetes Prevention Program, there was a 16% reduction in T2D incidence for every kilogram of weight lost (57). Lifestyle interventions in patients with prediabetes for 2-6 years have been shown to reduce the incidence of T2D by 27-67% in various ethnicities (58–60). Interventions with T2D medications (e.g., metformin, acarbose, rosiglitazone, pioglitazone, glargine, and semaglutide) for 1.5-6 years have reduced the incidence of T2D between 20-72% (58, 61). Likewise, anti-obesity medications taken for 1.2-4 years reduced the incidence of T2D between 19-79% (62).

In 2017, an expert group recommended using FINDRISC as a screening tool to detect impaired glucose metabolism in Latin America (63); this recommendation was included in some T2D clinical practice guidelines (CPG) in the region. Local validation of the FINDRISC's cutoff was reported in 2019 and proposed for the T2D CPG in Venezuela (16). CPGs from Colombia (64, 65), Brazil (66), Ecuador (67), Uruguay (68), Mexico (69), Argentina (70), and the Diabetes Latin American Association (ALAD) (71) have all adopted the recommendation of using FINDRISC for T2D screening, whereas the CPG in Peru (72) and Chile (73) have not. Criticisms of incorporating FINDRISC as part of organized screening argue that the downstream costs due to further testing and medication may not be justified. The implications of the lower specificity will result in unnecessary tests. Still, we assume that these additional costs are estimated to be much lower than the future treatment costs of the complications of an undiagnosed diabetes patient. Moreover, similar to the narrative about prediabetes and the development of the DBCD model, subsequent actions should be limited to simple diagnostics (FBG, OGTT, and/or A1C) and lifestyle interventions, reserving pharmacotherapy and procedures for guideline-directed management (74).

Internet coverage varies among the different Latin American countries. A limited access to the internet may affect equity in T2D screening in different regions of a country. With an average internet access rate of 68.8 percent in Latin America, the subregion of South America had the highest online access, with around 75 percent of its population having access to the web (75). However, access to the web has been shown to increase during the last decade and we believe that strategies like the one described in this manuscript may be used as a very cost-effective tool to screen people at high risk of diabetes. Finally, using artificial intelligence programs included in META (Instagram, Facebook, and WhatsApp), a direct response ad may be used to reach vulnerable and disadvantaged populations (older, low socioeconomic status, lower educational status). Online diabetes risk tools should be made available at institutions, organizations, and governmental agencies, as well as other primary healthcare settings working in T2D screening and prevention and be part of formal T2D preventive care programs. E-Health risk screening programs might facilitate the follow-up of T2D high-risk patients since their data may be available to the health system.

The strengths of the present study are related to the large-scale organized infrastructure, expedient implementation across diverse populations in Latin America and the Caribbean, and the use of social media platforms. Limitations are related to the self-reported nature of information collected and the associated potential bias. Specifically, people tend to underestimate reported anthropometric measures and overestimate reported healthy lifestyles. Also, since the FINDRISC is a prognostic tool, no inferences can be made about the true, overall prevalence of T2D or glucose metabolism disorders. A positive FINDRISC requires confirmation by diagnostic testing. In addition, this study consists of a non-probabilistic sample, so the results cannot be generalized to the overall population, thus limiting external validity. Lastly, the asymmetric distribution of the studied population in the region limits comparability and generalizations across the individual countries. In fact, the results could have also been confounded by the younger median ages of certain populations since they would more likely use social networks and eHealth technologies.

This study has important clinical and public health implications. The detection of early stages of DCBD (i.e., insulin resistance and prediabetes) by FINDRISC provides a screenshot of non-modifiable and modifiable risk factors that can be used to assess risks for many different chronic disease states. Online diabetes risk tools should be made available at institutions, organizations, and governmental agencies, as well as other primary healthcare settings working in T2D screening and prevention and be part of formal T2D preventive care programs. As in this study younger people were more likely to use the on-line screening tool, it is important to develop strategies to include older populations as well that are less familiar with the use of social media and the web in general. Thus, future studies should focus on optimizing this process with population-based cohort studies that incorporate transculturalization of lifestyle interventions mitigating DBCD progression across all age groups.

## Data availability statement

The raw data supporting the conclusions of this article will be made available by the authors, without undue reservation.

## Author contributions

Conceptualization, MR, RN-M, NB; methodology, MR; plan analysis, RN-M, NB; writing - original draft preparation, RN-M, NB; writing - review and editing, RN-M, NB, AG, AA, JM; final review, RN-M, NB, AG, JM. RN-M and NB share first authorship. All authors contributed to the article and approved the submitted version.
